# Effectiveness of virtual reality in nursing education: a systematic review and meta-analysis

**DOI:** 10.1186/s12909-023-04662-x

**Published:** 2023-09-28

**Authors:** Kai Liu, Weiwei Zhang, Wei Li, Ting Wang, Yanxue Zheng

**Affiliations:** 1 Nursing Department of Affiliated Hospital of Jining Medical University, 89# Guhuai Road, Rencheng district, Jining City, 272000 Shandong Province China; 2 Interventional Radiology of Affiliated Hospital of Jining Medical University, 89# Guhuai Road, Rencheng district, 272000 Jining City, Shandong China

**Keywords:** Virtual reality, Nursing education, Nursing students, Meta-analysis

## Abstract

**Objective:**

This study aims to assess the transformative potential of Virtual Reality (VR) has shown significant potential in transforming nursing education by providing immersive and interactive learning experiences. Our objective is to systematically evaluate and conduct a meta-analysizes on the impact effect of virtual reality technology in teaching nursing students.

**Methods:**

To achieve this, we conducted comprehensive computer searches on platforms including of PubMed, Web of Science, Wiley Online Library, Zhiwang database, Wanfang database, and China Biomedical Literature Service (SinoMed), were conducted to collect randomized controlled trial studies on the use of virtual reality’s technology for teaching nursing students built up to until March 2023., and the Cochrane Furthermore, the quality of the included literature was assessed evaluated using the quality evaluation criteria specified for randomized controlled trial studies within the Cochrane provided in the evaluation handbook manual. In addition, a meta-analysis was performed using Review Manager 5.3 software.

**Results:**

The aggregate outcomes from a total of 12 randomized controlled trials, encompassing including 1167 students, indicate were included. Meta-analysis results showed that virtual reality technology significantly enhances could better improve nursing students’’ theoretical knowledge [(SMD = 0.97, 95% CI [0.48, 1.46], p < 0.001)], practical skills (SMD = 0.52, 95% CI [0.33, 1.46], p < 0.001), skill retention, (SMD = 0.52, 95% CI [0.33, 0.71], p < 0.001), and satisfaction levels (SMD = 1.14, 95% CI [0.85, 1.43], p < 0.001), in comparison with traditional or alternative teaching methodologies. However, no statistically significant impact was observed on the enhancement of critical thinking skills (SMD = 0.79, 95% CI [-0.05, 1.64], p = 0.07) among nursing students.

**Conclusion:**

Our findings underscore that compared to conventional teaching methods, virtual reality offers superior potential in advancing nursing students’ theoretical knowledge, practice proficiencies, and overall satisfaction, while not yielding a significant advantage in enhancing critical thinking skills. The incorporated literature consisted exclusively of randomized controlled trials, albeit a subset of these studies omitted descriptions of the allocation concealment strategy.

## Introduction

Virtual reality (VR) technology, stemming from mathematical reasoning and scientific experimentation, assumes a pivotal role as a universal and strategic tool for comprehending, reshaping, and innovating the tangible world [1, 2]. VR involves creating a computer-generated simulation of a three-dimensional image or environment that individuals can interact with as if it were real or physical. Thus, this interaction is achieved through specialized electronic equipment, such as a helmet with an integrated screen or gloves equipped with sensors. This holds within the evolving landscape of education reform and heightened aspirations for elevated higher education standards. Significantly accentuating this trajectory, VR’s educational potential is thrust into the spotlight [3], propelling it into a burgeoning realm of investigation with expansive practical applications. Notably in the domain of nursing education, this technology garners increasing attention from medical education scholars who seek to curtail teaching expenses and mitigate instructional hazards while upholding pedagogical excellence [4–6].

Amid the rapid evolution of information technology, novel advancements like VR are ushering in a fresh pedagogical approach to nursing education [7]. This technique engenders an immersive, interactive encounter for nursing students, replicating authentic clinical scenarios and furnishing them with robust hands-on training experiences, all while circumventing direct patient engagement. This not only economizes the valuable time of clinical nursing professionals but also mitigates the predicaments associated with conventional patient interaction in pedagogy [8], thereby addressing the dearth of clinical educational resources. Nursing students interact with VR through immersive experiences that simulate real-life clinical scenarios. Using specialized VR equipment, such as VR headsets or gloves with sensors, nursing students can engage with virtual patients, medical equipment, and healthcare environments.

This allows them to practice clinical skills, make decisions in simulated patient care situations, and explore various medical scenarios. VR technology enables nursing students to actively participate in realistic learning experiences, enhancing their understanding of theoretical concepts and providing hands-on training in a safe and controlled environment. Besides, the interactive dimension inherent to VR technology augments the didactic process, bestowing nursing students with an intuitively enriched learning experience [9]. Through its immersive interactivity, VR effectively eradicates the dullness of conventional teaching, and rigid teaching methodologies, kindling greater scholarly enthusiasm among nursing students and markedly improving their operational skills [10]. By proficiently role-modeling clinical environments, VR fosters a profound sense of engagement, inherently nurturing nursing students’ professional self-conception [11], strengthening their sense of commitment and calling, and galvanizing autonomous knowledge exploration. Moreover, the interactive operability at the core of VR fundamentally amplifies the intrinsic impetus for nursing students to actively pursue learning [12].

Jang et al. [13] demonstrated that the interactive and operational attributes of VR outperform 3D videos in enhancing students’ comprehension and assimilation of knowledge. Within the realm of nursing education, there exists a significant challenge in devising a curriculum that encompasses both depth and breadth, rectifying the limitations intrinsic to conventional pedagogy. This challenge seeks to seamlessly guide nursing students in the transition from fragmented textual knowledge to its clinical application, concurrently conserving invaluable clinical teaching resources and nurturing adept nursing practitioners [14].

Virtual reality (VR) is the use of computer technology to create an interactive three-dimensional (3D) world, which gives users a sense of spatial presence. In nursing education, VR has been used to help optimize teaching and learning processes. In the contemporary landscape of nursing education reform, this stands as a crucial and intricate juncture demanding resolution. Consequently, a symbiotic fusion of nursing education with the ever-evolving era is indispensable for nurturing adaptable nursing professionals capable of propelling the progress and expansion of both nursing education and clinical practice. As the trajectory of nursing education evolves, the integration of VR instruction emerges as a definitive trend [15], yet it remains in its nascent exploratory phase, necessitating substantial engagement from nursing educators to chart a scientifically sound path toward effective VR nursing education.

Hence, this study meticulously reviewed the existing literature concerning the integration of VR into nursing education curricula, with a focus on both current and prospective nursing educators. Employing a systematic evaluation and subsequent meta-analysis, we aimed to discern optimal approaches in nursing student instruction, by identifying essential attributes of nursing education and furnishing a guiding trajectory for future research about virtual reality-infused nursing educational programs.

## Methods

### Study design

This study employed “The Preferred Reporting Items for Systematic Reviews and Meta-Analyses”, otherwise known as the principles of the PRISMA statement in the reporting of the meta-analysis [16].

### Search strategy

**Fig. 1:**
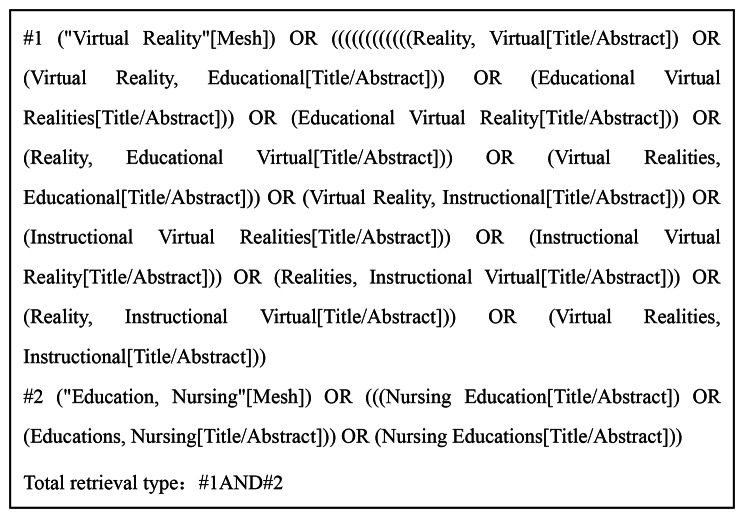
Search strategy

We conducted computer-based searches across multiple databases including PubMed, Web of Science, Wiley Online Library, Zhiwang database, Wanfang database, and China Biomedical Literature Service (SinoMed). The search spanned from the establishment of each database up to March 2023, employing a search strategy that combined both free terms and subject descriptors. Equally, by employing literature tracing techniques, we located pertinent studies. On top of that, the search strategy encompassed a fusion of free terms and subject descriptors. Within the English databases, search terms include “virtual reality/patient simulat*/virtual patient*/virtual simulation” and “education, nursing /nurs*education/education of nursing.” As an illustrative instance, the search strategy utilized for PubMed is outlined in Fig. 1.

### Inclusion and exclusion criteria

#### Inclusion criteria

Adhering to the population, intervention, control, outcomes, and study design (PICOS) principles, we methodically screened the literature based on the following criteria: (1) study population (P): nursing students; (2) intervention (I): experimental groups in the studies that employed VR technology for instruction, implementing the four essential components delineated by Sherman and Craig (virtual world, immersion, sensory feedback, interactivity) to establish varying degrees of immersion through diverse VR platforms [17]; (3) control measures (C): control groups were exposed to conventional pedagogical techniques (comprising classroom lectures, demonstrations, model-based instruction, etc.) or non-VR simulation scenarios (encompassing high-fidelity/low-fidelity simulation, mannequin simulation, etc.); (4) outcomes (O): evaluated outcome indicators, which includes theoretical knowledge scores, practical skills scores, satisfaction levels, and critical thinking abilities; (5) study design (S): exclusively encompassing randomized controlled trials.

#### Exclusion criteria

We excluded the following types of literature during the selection process: (1) conference papers, abstracts, and catalogs; (2) duplicate publications; (3) literature containing errors, incomplete study data, or being inaccessible for comprehensive analysis; (4) literature with inaccessible full text; (5) literature not published in English or Chinese; (6) Other types of literature besides experimental studies.

### Study selection and data extraction

Two researchers conducted an independent screening of the literature based on the inclusion and exclusion criteria. Initially, they reviewed the titles and abstracts of the literature, excluding those that did not meet the inclusion criteria. Subsequently, a meticulous assessment of the full texts was undertaken for the remaining literature, leading to their inclusion upon alignment with the set criteria. Ultimately, the literature that adhered to the inclusion criteria was definitively identified. In instances of discrepancies, a third researcher was consulted to facilitate the decision-making process, and any information gaps were addressed by reaching out to the original authors whenever feasible. When confronted with duplicate publications, precedence was given to the Chinese literature.

Following the identification of the literature, two researchers autonomously undertook the task of data extraction. They meticulously extracted data from the selected studies in alignment with the prescribed data extraction protocols as outlined in the Cochrane Handbook for the Evaluation of Intervention Systems [18]. Furthermore, the extracted information encompassed a range of elements including authors, year of publication, country, study population, number of participants, and number of interventionists.

Correspondingly, we adhered to the risk of bias assessment of the principles for Randomized Controlled Trials (RCT) as prescribed by the Cochrane Collaboration Network Handbook on Systematic Evaluation of Interventional Studies, version 5.1. Two investigators autonomously conducted evaluations of the included RCTs, while any disparities were resolved through deliberation or consultation with a third investigator when necessary.

### Study quality and risk of bias assessment

Two investigators independently conducted assessments in accordance with the evaluation criteria outlined in Cochrane Evaluation Manual 5.1.0 [19]. In instances where discrepancies arose, a third investigator was consulted to facilitate resolution through discussion or consultation. The assessment encompassed the following items: ①generation of randomized sequences; ②allocation scheme concealment; ③blinding of investigators and subjects; ④blinding of assessors; ⑤completeness of data; ⑥selective reporting; and ⑦other biases. Thus, the classification of risk levels for each element was divided into high risk, low risk, and unclear. This study, therefore, elucidates the rationale behind each judgment by the assessment framework of the Review Manager 5.3 program.

### Statistical methods

We conducted the meta-analysis of the included literature utilizing Review Manager 5.3 software and Endnote software. Continuous variables were elucidated through mean difference (MD), standardized mean difference (SMD), and a 95% confidence interval (CI). Statistical significance and differences between the experimental and control groups were established at P < 0.05. Besides, we assessed the heterogeneity of the meta-analysis results utilizing the I^2^ quantitative test. A value of I^2^ < 50% indicated low heterogeneity, enabling the selection of a fixed-effect model. Conversely, an I^2^ value ≥ 50% denoted high heterogeneity, warranting the adoption of a random-effect model. For I^2^ values exceeding 75%, significant heterogeneity within the meta-analysis results was ascertained.

## Results

### Characteristics of the study population (study selection)

**Fig. 2:**
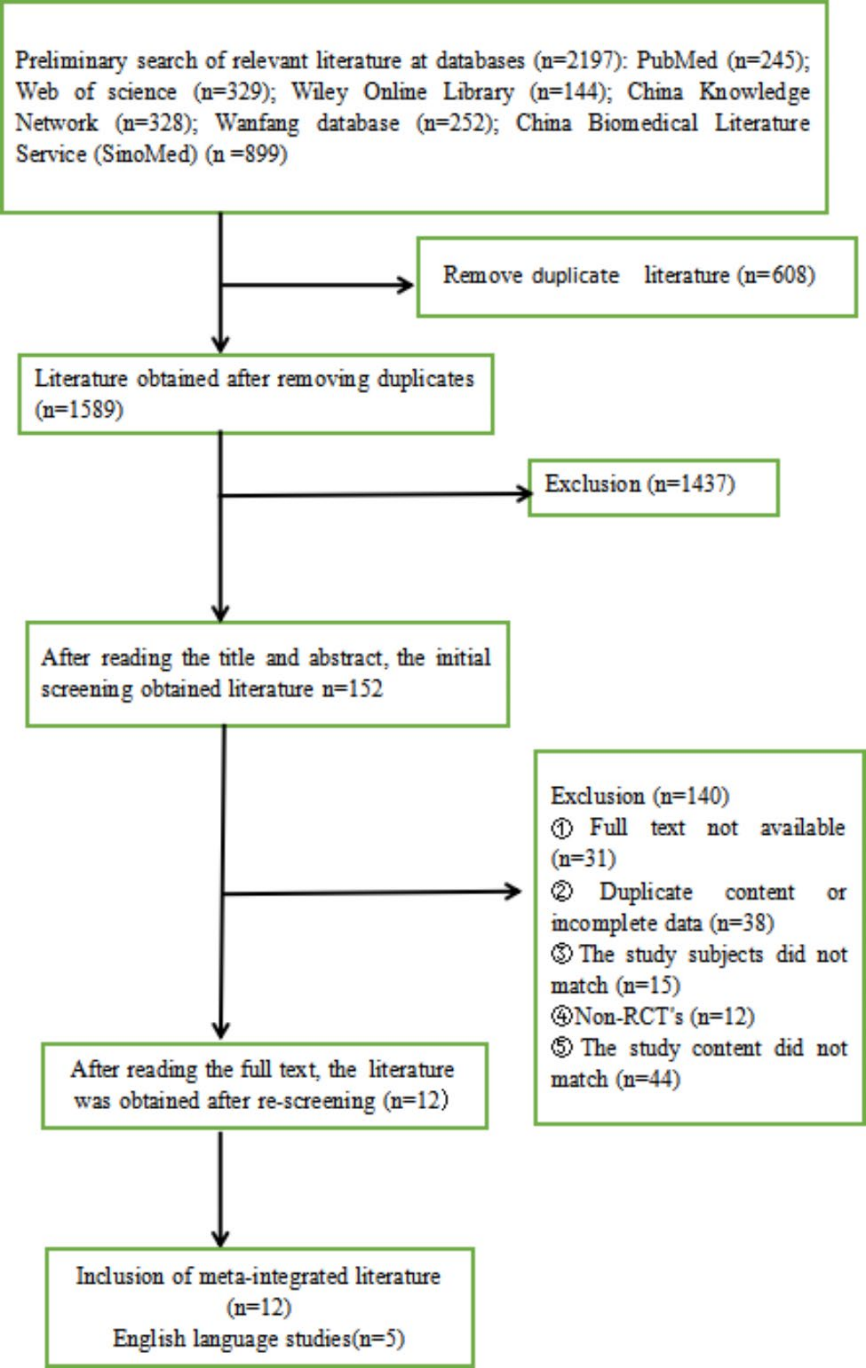
Literature screening process and result

A computer search of PubMed, Web of Science, Wiley Online Library, Zhiwang database, Wanfang database and China Biomedical Literature Service (SinoMed) retrieved 2179 papers; titles and abstracts were reviewed and 152 articles were selected considering the inclusion and exclusion criteria. After excluding 140 papers that did not meet the inclusion criteria, 12 papers were finally selected [20–31]. As shown in Fig. 2.

### Characteristics of research on educational interventions using virtual reality for current and future health personnel (research characteristics)

This study includes general characteristics of 12 studies of educational interventions using virtual reality; detailed information is provided in Table 1 In terms of study design characteristics, all 12 studies were randomized controlled studies. The number of study participants was 585 for the experimental group and 582 for the control group, for a total of 1167 participants.

**Table 1:** Characteristics of selected 12 studies included in the systematic review

Author (year)	country	Type of participant	Number of participants			Interventions		Outcomes
			Total	E	C	E	C	
José Miguel Padilha (2019)	Portuguese	nursing students	42	21	21	VR	traditional education	Learningsatisfaction;Knowledge;Self-efficacy perception
Hanna Lee (2022)	South Korea.	fourth-year students across nursing colleges	60	30	30	VR	traditional education	Self-efficacy; clinical reasoning capacity; learning satisfaction; Knowledge
Khaild AL-MugheedI (2022)	North Cyprus	nursing students	122	63	59	VR + online education	traditional education	Knowledge; Attitude
Mi Yu (2021)	Korea	nursing students	50	25	25	VR	routine education	knowledge ; self-effycacy ; learning satisfaction
Hsiang-Ying Chan (2020)	China	nursing students	77	38	39	VR	education documents	Knowledge; Attitude
Ae-Ri Jung (2022)	Korea	nursing students	60	30	30	virtual reality (VR) nursing education program (VRP)	traditional nursing education program (VRP)	Knowledge;Attitude;Satisfaction;Motivation
PingWang (2020)	China	nursing students	125	62	63	Immer VR	Non-immer VR	Knowledge ;Practical skills, reasoning ability
Tianxiang Yuan (2019)	China	nursing students	116	58	58	“flipped classroom” teaching model based on virtual reality animation micro course	traditional education	Knowledge ;Practical skills
Nan Cao (2021)	China	nursing students	90	45	45	VR	traditional education	Knowledge ; Practical skills, Critical thinking; clinical communication skills
Liping Li (2017)	China	nursing students	100	50	50	VR	traditional education	Knowledge ;Practical skills
Xiaoyan Wang (2023)	China	nursing students	245	123	122	VR	traditional education	Self-directed learning skills; critical thinking
Hongmei Zhao (2022)	China	nursing students	80	40	40	VR	traditional education	Knowledge;Self-directed learning skills;critical thinking

E = Experimental group; C = Control group; VR = Virtual reality;RCT = randomized controlled trial

### Methodological quality assessment of intervention studies

**Fig. 3:**
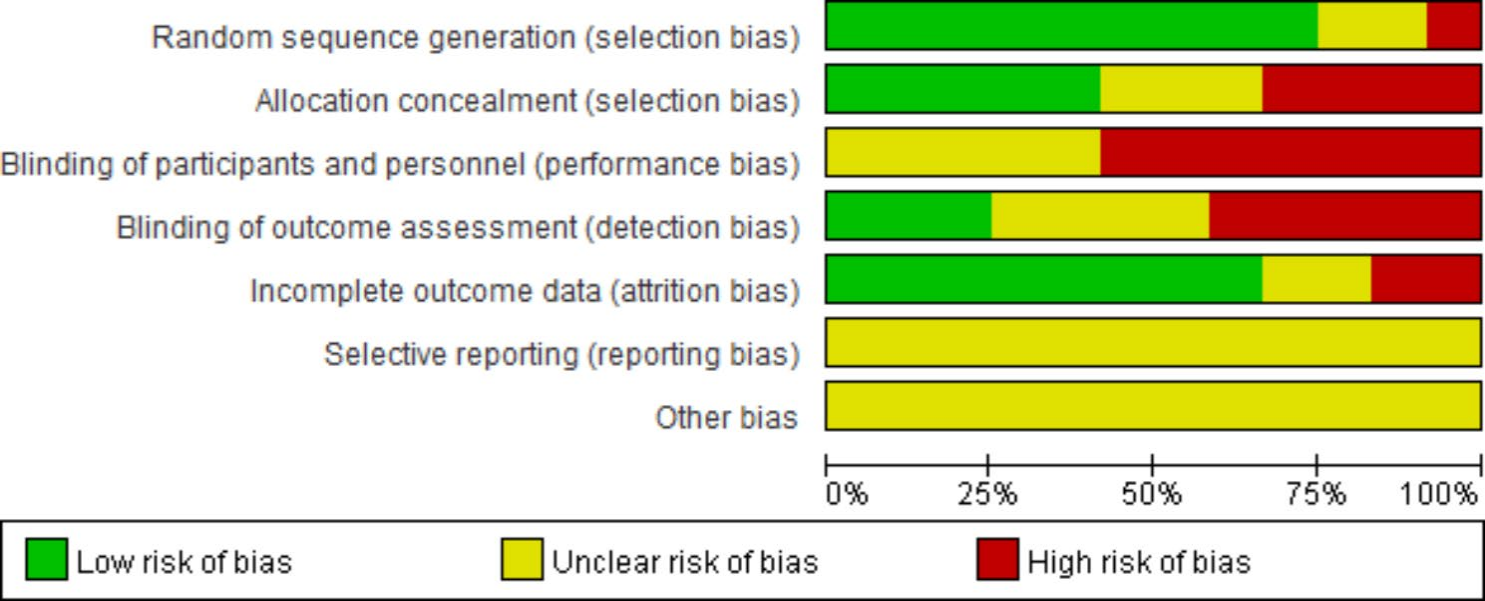
Overall risk of bias analysis of included studies

**Fig. 4:**
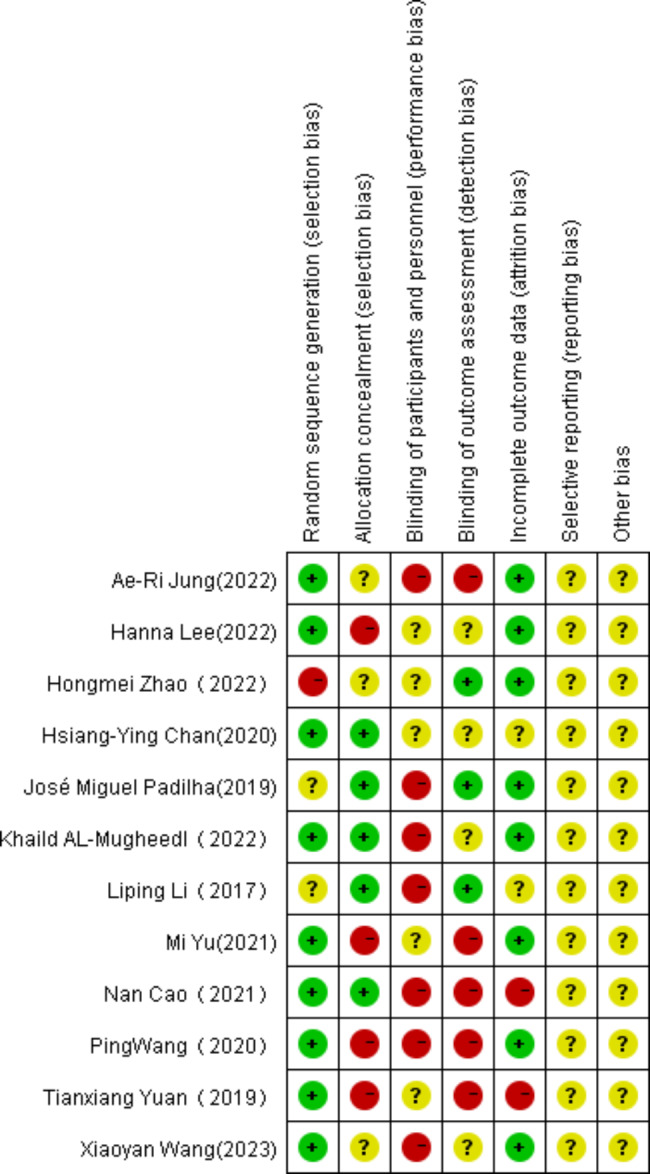
Risk of bias analysis of each included study

The risk of bias assessment is summarized in Fig. 3 below. 9 of the 12 studies described detailed information related to randomization, and 7 studies lacked clarity regarding allocation concealment. The detailed risk of bias assessment is shown in Fig. 4.

### Meta-analysis results

#### Theoretical knowledge

**Fig. 5:**
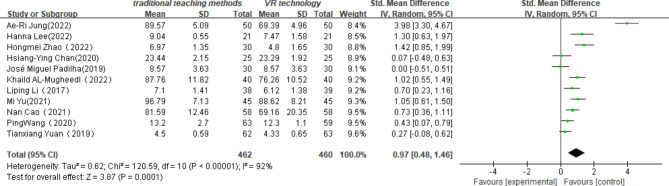
Impact of VR technology on nursing students’ theoretical knowledge

Eleven studies^[20–25,27−31]^evaluated the effectiveness of VR technology in theoretical knowledge levels. The results showed a high heterogeneity of the included studies (p < 0.001, I2 = 92%), so a random effects model was used. The combined results showed that the use of VR technology was effective in improving students’ theoretical knowledge compared to other traditional nursing teaching methods (SMD = 0.97, 95% CI [0.48, 1.46], P < 0.001, Fig. 5) .

#### Practical skills

**Fig. 6:**
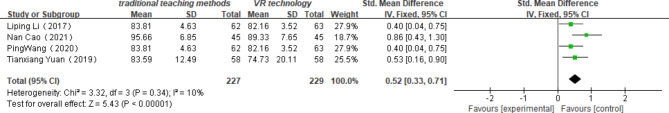
Impact of VR technology on nursing students’ practice skills

Four studies [24, 28, 29, 31]evaluated the effectiveness of VR technology in practice skills. The results showed that there was no heterogeneity in the included studies (P = 0.34, I2 = 10%), so a fixed-effects model was used. The combined results showed a statistically significant difference compared to other traditional nursing teaching methods (SMD = 0.15, 95% CI [-0.21, 0.51], P < 0.001, Fig. 6).

#### Satisfaction

**Fig. 7:**
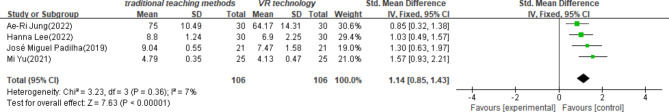
Impact of VR technology on academic satisfaction of nursing students

Four studies [20, 21, 23, 25]evaluated the effect of VR technology on nursing teaching satisfaction, and the results showed that there was no heterogeneity in the included studies (P = 0.36, I^2^ = 7%), so a fixed-effects model was used. The combined results showed a significant difference in the improvement of satisfaction with nursing education with VR technology compared to the control teaching modality (SMD = 1.14, 95% CI [0.85, 1.43], P < 0.001, Fig. 7).

#### Critical thinking

**Fig. 8:**
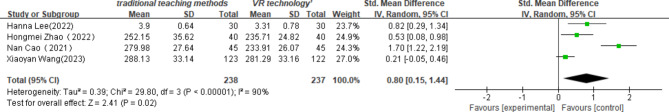
Impact of VR technology on nursing students’ critical thinking

Three studies [26, 30, 31]evaluated the effect of VR technology on the application of critical thinking among nursing students, but the results showed high heterogeneity in the included studies (P < 0.001, I^2^ = 93%), so a random effects model was used. The combined results showed no significant difference in the improvement of satisfaction in nursing education with VR technology compared to the control teaching modality (SMD = 0.79, 95% CI [-0.05, 0.46], p = 0.07, Fig. 8).

## Discussion

### The effect of the application of VR technology on nursing students

The results of the aforementioned meta-analysis revealed that the utilization of VR technology in nursing student education yielded more effective enhancement in theoretical knowledge in comparison to other conventional teaching methods, and this distinction achieved statistical significance (P < 0.05). Contemporaneously, the theoretical component of nursing education stands as a crucial underpinning for nurses to translate their knowledge into clinical competence, constituting an integral part of nursing instruction. In a bid to bolster the teaching effectiveness of the theoretical course, nursing educators have incorporated virtual reality technology. Similarly, West Chavez et al. [32] demonstrated how VR technology increased student engagement by immersing them in realistic learning experiences that closely mirrored their real environment.

Moreover, a qualitative exploration into VR technology’s integration within nursing education [33] suggests that amalgamating VR technology with conventional nursing pedagogy enables students to interact with objects within a specific virtual teaching environment, engendering equivalent sentiments and experiences as in the real environment. This immersion empowers learners to fathom what they have imbibed and how to practically apply that knowledge. Furthermore, aligned with Kolb’s experiential learning model [34], nursing students glean insights from their virtual world experiences akin to real-life occurrences, thereby garnering more immediate and enduring outcomes. This explains the observed escalation in theoretical knowledge. According to Pei Ning Woon et al. [35] ,Virtual reality may be a viable teaching strategy to improve knowledge acquisition, but it is presently suitable for supplementing conventional teaching methods. However, the incorporation of VR technology into nursing instruction not only captivates nursing students’ dedication to learning but also fortifies their knowledge and skills, serving as a foundational requisite for propelling nursing students’ transition from knowledge-based to competence-driven paradigms.

### VR technology effect on the practical skills of nursing students

In comparison with conventional or alternative nursing education methodologies, the application of VR technology in teaching exhibits a disparity in nursing practical skills (P > 0.05). That said, virtual simulation technology distinctly elucidates operational intricacies, facilitating accelerated and comprehensive knowledge assimilation among students, thereby augmenting instructional efficacy. A pertinent example stems from Hong Kong Polytechnic University’s successful integration of virtual reality technology in nasogastric tube placement training, attesting to the technology’s safety, flexibility, and interactivity advantages that accelerates the learning curve for this procedure [36].

Consistent with prior studies, Jefferson (2022) devised a high-fidelity simulation (HFS) course, whereby participants exemplified heightened learning retention and enhanced practical aptitude levels [37]. Consequently, future endeavors should prioritize refining the technical effectiveness of VR teaching environments, as well as enhancing the transference of acquired practical skills from virtual to clinical settings. In addition, empowering nursing students to engage with patients within virtual environments considerably enhances their clinical technique comprehension and enables them to navigate clinical scenarios during practice. Congruently, certain techniques unfeasible for real patients can be practiced on virtual patients, thereby streamlining the transformation process from theory to practice, student to practitioner, and classroom to clinical settings. This redresses inherent limitations within traditional practical training approaches, subsequently boosting nursing students’ learning efficiency [38].

### VR technology’s improvement effect on nursing students’ academic satisfaction

VR technology imbues learning with vividness and imagery, exuding a potent contagious influence that substantially heightens nursing students’ perceptual acuity, enthusiasm, and active engagement in learning, effectively positioning them at the center of the learning process. When applied to nursing instruction, VR amplifies the liveliness of educational content, fostering augmented interest in independent learning among nursing students, and catalyzing a shift from passivity to activeness in their journey of intellectual voyage. Apart from that, this technology propels students beyond the mere exercise of operational skills within virtual contexts; rather, it serves as a platform where they refine not only operational proficiencies but also their clinical reasoning, decision-making acumen, and aptitude for troubleshooting practical clinical problems [39]. At the same time, VR offers a robust milieu for collaborative learning, promoting teacher-student interactions. Through group discussions and cooperative endeavors facilitated by VR, educators, and learners deepen their problem analysis and critical thinking, honing their cognitive capacities, and inadvertently enhancing students’ prowess in collaborative communication. This progressive enhancement of collaborative communication inadvertently bolsters the academic contentment of nursing students [40].

### The effect of VR technology on the level of nursing students’ critical thinking

The findings of this study revealed a lack of statistically significant difference (P > 0.05) in the improvement of critical thinking with in nursing education through the implementation of VR technology. This outcome is chiefly attributed to the paucity of research on the relationship between virtual learning environments and the cultivation of critical thinking skills among college students. Also, the obscurity surrounding the determinants influencing college students’ critical thinking development within virtual learning environments, the intricate processes underlying each determinant’s impact, and the varying degrees of influence exerted by these determinants, remain unresolved.

By the same token, capitalizing on the rapid advancements in virtual reality technology, researchers have turned their attention towards harnessing virtual learning environments to foster and nurture college students’ critical thinking prowess. Notably, Kandi et al. (2020) conducted an experimental inquiry that unveiled that architecture students immersed in a virtual learning environment exhibited enhancements in their design, review, and innovation skills. Furthermore, the study ascertained that a virtual reality game design simulator empowered students to pinpoint design errors more effectively and subsequently excel in critical thinking tests [41]. In like manner, Kang (2020) developed a virtual reality nursing course and found that it facilitated students’ critical thinking development and independent learning skills [42]. Nonetheless, the utilization of virtual learning environments encounters challenges such as heightened cognitive load and information disorientation. Consequently, the meticulous design of virtual learning environments to maximize the development and augmentation of college students’ critical thinking capacities becomes paramount.

### Limitations and prospects of this study

The conclusions drawn from this study are based on a high-quality randomized controlled trial, significantly elevating the strength of its evidentiary foundation in contrast to cohort studies. This robust evidence, synonymous with evidence-based medicine, lays a foundational bedrock for the prospective application of virtual reality technology within nursing education. Nevertheless, this meta-analysis has certain limitations: (1) The incorporated literature consisted exclusively of randomized controlled trials, albeit a subset of these studies omitted descriptions of the allocation concealment strategy; (2) Disparities in intervention types and assessment methods for outcome indicators among the included studies might have potentially influenced the eventual aggregated outcomes; (3) This investigation limited its scope to Chinese and English literature, potentially introducing an influence on the study outcomes. Consequently, to further establish the efficacy of psychotherapy in treating depression and anxiety among college students, subsequent stages necessitate the design of more rigorous, multicenter, and large-scale randomized controlled trials.

## Conclusion

The outcomes of this meta-analysis demonstrate that VR technology can be more effective in improving nursing students’ knowledge of nursing teaching skills, practical nursing teaching aptitude, and academic contentment. Nevertheless, no notable superiority of VR technology was observed in enhancing nursing students’ critical thinking abilities. This could potentially stem from variations in intervention delivery, assessment methodologies, study participants, and research schemes. Consequently, educators must reorient their teaching paradigms, reinforcing the significance of virtual reality technology, and proactively integrating advanced technological tools for educational advancement. Based on the aforementioned points, VR technology stands poised to emerge as a pivotal breakthrough in the future of education, ushering in far-reaching impacts on the evolution of pedagogical methodologies in nursing education.

In conclusion, this study demonstrates the potential of VR technology to enhance nursing education by improving theoretical and practical knowledge as well as academic satisfaction among nursing students. However, the absence of a significant advantage in enhancing critical thinking skills through VR interventions suggests the need for further investigation into the design of VR-based learning environments tailored to fostering critical thinking. Despite the rigorous methodology applied in this study, limitations include variations in intervention types, assessment methods, and subject characteristics across the included studies. To address these limitations, future research should focus on refining VR interventions for nursing education, considering the specific components that effectively promote critical thinking, and conducting multicenter studies with larger sample sizes to provide more robust evidence of VR’s impact on nursing education outcomes.

## Data Availability

All data generated or analyzed during this study are included in this published article.

## Abbreviations

CNKI: China National Knowledge Infrastructure
MD: Mean difference
PRISMA: The Preferred Reports for Systematic Reviews and Meta-Analyses
RCT: Randomized controlled trial
SinoMed: China Biomedical Literature Service
SMD: Standardized mean difference.
VR: Virtual reality
